# Archaeal DNA replication initiation: bridging LUCA's legacy and modern mechanisms

**DOI:** 10.3389/fmicb.2025.1561973

**Published:** 2025-02-19

**Authors:** Rémi Dulermo

**Affiliations:** Univ Brest, Ifremer, Biologie et Ecologie des Ecosystèmes Marins Profonds (BEEP), Plouzané, France

**Keywords:** LUCA, DNA replication, homologous recombination, archaea, cyanobacteria, *Deinococcus*, RDR, R-loop

## 1 Introduction

DNA replication is an essential process enabling the duplication of DNA before cell division. This process typically starts at origins of replication (*ori*), which are specific sequences (except in higher eukaryotes, where they are not very well defined and correspond to a broad chromosomal region where DNA replication preferentially happens) recognized by an initiator protein complex, leading to the unwinding and opening of DNA. In bacteria, the initiator is DnaA (Rashid and Berger, 2024), while in archaea and eukaryotes, it is initiated by Orc/Cdc6 proteins (Orc1/Cdc6 in Archaea and Orc1-6 and Cdc6 in eukaryotes; Bell and Dutta, 2002; Matsunaga et al., 2007; Majerník and Chong, 2008; Ojha and Swati, 2010). Bacteria typically possess a single *ori* (Gao, 2015), whereas eukaryotes can have hundreds to thousands (Hu and Stillman, 2023; Tian et al., 2024). In archaea, the number of origins of replication varies between species, ranging from one to four (Myllykallio et al., 2000; Lundgren et al., 2004; Duggin et al., 2008; Farkas et al., 2011; Pelve et al., 2012; Hawkins et al., 2013). Then, initiator proteins (DnaA or Orc1/Cdc6) recruit replicative helicase (DnaB for bacteria, Mcm in archaea and MCM complex in eucaryotes) that unwinds DNA and replication begins with the loading of the other components (primase, polymerases etc.).

Positioned evolutionarily between bacteria and eukaryotes (Dombrowski et al., 2021; Imachi et al., 2020), archaea offer a unique vantage point for understanding the mechanisms that drive cellular life. While archaea were initially thought to share replication strategies with bacteria due to their prokaryotic nature, studies have shown that they employ a hybrid system incorporating features from both bacterial and eukaryotic replication processes. These microorganisms possess genes in operons and circular genomic DNA like bacteria, but their DNA metabolism is more closely related to that of eukaryotic cells (Edgell and Doolittle, 1997; Raymann et al., 2014). This evolutionary bridge makes archaea fascinating subjects for scientific inquiry, offering insights that could reshape our understanding of molecular biology.

The study of DNA replication in archaea has garnered increasing attention over the past decade, especially since it was shown that archaea can live without *ori* (Hawkins et al., 2013). Recent studies have demonstrated that archaea can use multiple origins of replication with different levels of activity, and that homologous recombination plays an important role in their DNA replication (Hawkins et al., 2013; Gehring et al., 2017; Mc Teer et al., 2024; Liman et al., 2024). This article aims to explore the latest findings on archaeal DNA replication, focusing on key studies by Hawkins et al. (2013), Mc Teer et al. (2024), and Liman et al. (2024). These works not only challenge traditional views but also highlight the complexity and flexibility of archaea.

## 2 Evolutionary perspective on DNA replication

DNA replication is fundamental to life, yet the processes employed by archaea defy simple classification. Historically, scientists grouped archaea with bacteria due to their prokaryotic structure, but Carl Woese and coworkers revolutionized this perception by demonstrating the existence of three distinct domains of life (Fox et al., 1977). Although it is still debated whether archaea are the ancestors of eukaryotes (Imachi et al., 2020; Dombrowski et al., 2021; Da Cunha et al., 2022), certain aspects of their biology remain intriguing. Research from Hawkins et al. (2013), and more recently Gehring et al. (2017) and Mc Teer et al. (2024), reveals a more complex reality. Archaea not only use a combination of bacterial-like and eukaryotic-like DNA replication mechanisms, but they might also retain even more ancient processes. This suggests that archaea might represent an evolutionary bridge linking ancient and modern DNA replication systems. One of the most striking discoveries is the role of homologous recombination in DNA replication in the Euryarchaeota *Haloferax volcanii* (Hawkins et al., 2013). Mc Teer et al. (2024) and Liman et al. (2024) demonstrated that RadA is involved in DNA replication since reducing RadA expression increases *ori* utilization in two other Euryarchaeota, *Thermococcus barophilus* and *Thermococcus kodakarensis*, respectively. These findings reveal that recombination-dependent replication (RDR) is used by some archaea and partly or entirely ensures proper DNA replication. However, not all archaea can live without *ori*, nor do they all use RDR (Samson et al., 2013; Yang et al., 2015). RDR relies on the formation of a D-loop through strand invasion by homologous recombination, which then serves as a platform to initiate DNA replication. RDR was firstly described in T4 phage (Mosig, 1998; Kreuzer, 2000; Malkova and Ira, 2013). This phage has a special DNA replication system, using R-loop (a RNA invades double strand DNA to initiate DNA replication) and RDR (Miller et al., 2003). Similar mechanisms as RDR were also described in *Escherichia coli* (iSDR that used recombinase or cSDR that used R-loop) and in eukaryotes (BIR) but they are unable or weakly able to form colonies or replicate DNA without error (Michel and Bernander, 2014). Interestingly, it was shown that some Cyanobacteria are able to live without *dnaA* (Richter et al., 1998; Ran et al., 2010; Ohbayashi et al., 2016, 2020). Ohbayashi et al. (2016) suggested that multiple replication origins fire asynchronously in this strain to explain their results. This could, at least for some Cyanobacteria, be in accordance with the RDR found in archaea. Unfortunately, no study has yet investigated the importance of RecA, the bacterial recombinase, in Cyanobacteria. Such research is necessary to determine if Cyanobacteria use RDR or another unknown DNA replication initiation pathway. These findings suggest a shared evolutionary origin for RDR and related mechanisms, potentially dating back to LUCA (Last Universal Common/Cellular Ancestor).

In my opinion, these discoveries challenge the “classical” models of DNA replication. Archaea are not merely exceptions to the rule but are critical in refining our understanding of the fundamental processes shared by all life forms. Their replication systems blur traditional evolutionary boundaries and serve as living models of early cellular life.

## 3 Recombination before the origin of replication?

Forterre (2002, 2013) proposed that replication machineries, which differ between Bacteria and Archaea/Eucarya, could result from viral transfer in descendants of LUCA. Similarly, Koonin (2014) explained that a variety of replication strategies associated with respective molecular systems may have evolved in the primordial pool, with only some surviving in selfish elements. Two of these strategies were ultimately adopted by evolutionarily successful cellular life to form Bacteria and Archaea, and later Eukaryotes. Since the RecA/RAD51/RadA protein family shares a common ancestor dating back to LUCA (Lin et al., 2006; Chintapalli et al., 2013), this could explain why RDR or similar mechanisms, such as iSDR or BIR, are found in many organisms. Kowalczykowski (2000) proposed that RecA-like proteins provided a simple way to initiate DNA replication in primitive organisms.

Since, it has been shown that only the ATPase AAA+ domain of DnaA/Orc/Cdc6 proteins share a common ancestor dating to the same period (Iyer et al., 2003), it suggests that DnaA and Orc/Cdc6 proteins was obtained by a fusion gene (specific proteins that binds ori with the ancestor of the actual AAA+ ATPase domain of DnaA/Orc/Cdc6) independently in the descendant of LUCA that gave the ancestor of bacteria and archaea/eukarya respectively. Thus it is possible that only homologous recombination was present in LUCA. Genome of LUCA remains mysterious since it was proposed that it was composed of DNA (Forterre, 1999; Koonin et al., 2020) and now it has been suggested that LUCA could still have a RNA genome (Forterre, 2024). This hypothesis is based on non-homology of replicative polymerase, DNA helicases and primase in the archaea/eukarya and the bacteria and that actual universal DNA proteins worked on RNA such as Topo IA or that they were given by a virus (PCNA, RFC for example) in descendants of LUCA. To go further with this theory, an increasing number of studies have shown that RNA-binding proteins are involved in DNA repair (Bader et al., 2020; Klaric et al., 2021), and some proteins known to be involved in DNA repair are also known to bind RNA, such as BRCA1, KU, RPA, RAD52, RAD51, and RecA (Kirkpatrick et al., 1992; Kim et al., 1992; Yoo and Dynan, 1998; Keskin et al., 2014; Sharma et al., 2015; Thomas et al., 2023). For this review, and specifically for RecA/RAD51/RadA family proteins, it is tempting to think that the ancestor of these proteins—the RecA ancestor—already present in RNA-LUCA, could perform RNA repair and RNA replication, such as RecA/RAD51/RadA family proteins perform DNA repair and DNA replication in modern organisms. In other word, it might be the first simple system to replicate (and repair) RNA in LUCA and may be later to replicate DNA (in LUCA's descendants). This could actually support the view that the R-loop generated by RNA Polymerase (a simple one was present in LUCA) or by RecA ancestor (RecA is able to form R-loop (Kasahara et al., 2000) acted as an ancestral system for replication initiation. Then, *ori* and replicative initiator was taken by ancestor of bacteria and archaea/eukaryotes.

In another case, it is possible that one or two DNA viruses already carried the ancestors of the RecA/RadA/RAD51 protein family and/or DNA replication initiators, which were then transmitted to LUCA's descendants. Supporting this hypothesis, it was recently shown that origins of replication may originate from extrachromosomal genetic elements, as demonstrated by Robinson and Bell (2007) and Hawkins et al. (2013, the fourth *ori* of *H. volcanii* chromosome). This suggests that the replication origins seen in modern organisms may have evolved from mobile genetic elements absorbed and regulated by the cellular machinery of LUCA's descendants. Therefore, these elements, initially selfish and independent, may have been co-opted to create more efficient, centralized systems for DNA replication. Since recombination can be used by different organisms not only to repair DNA but also to restart or perform DNA replication, one particularly fascinating hypothesis is that homologous recombination may have preceded, or co-existed with, the emergence of *ori* as the primary method to initiate DNA replication. Some organisms retained this mechanism (archaea, cyanobacteria?) or a similar one (bacteria and eukaryotes) to replicate their genomes under specific conditions.

**Figure 1:**
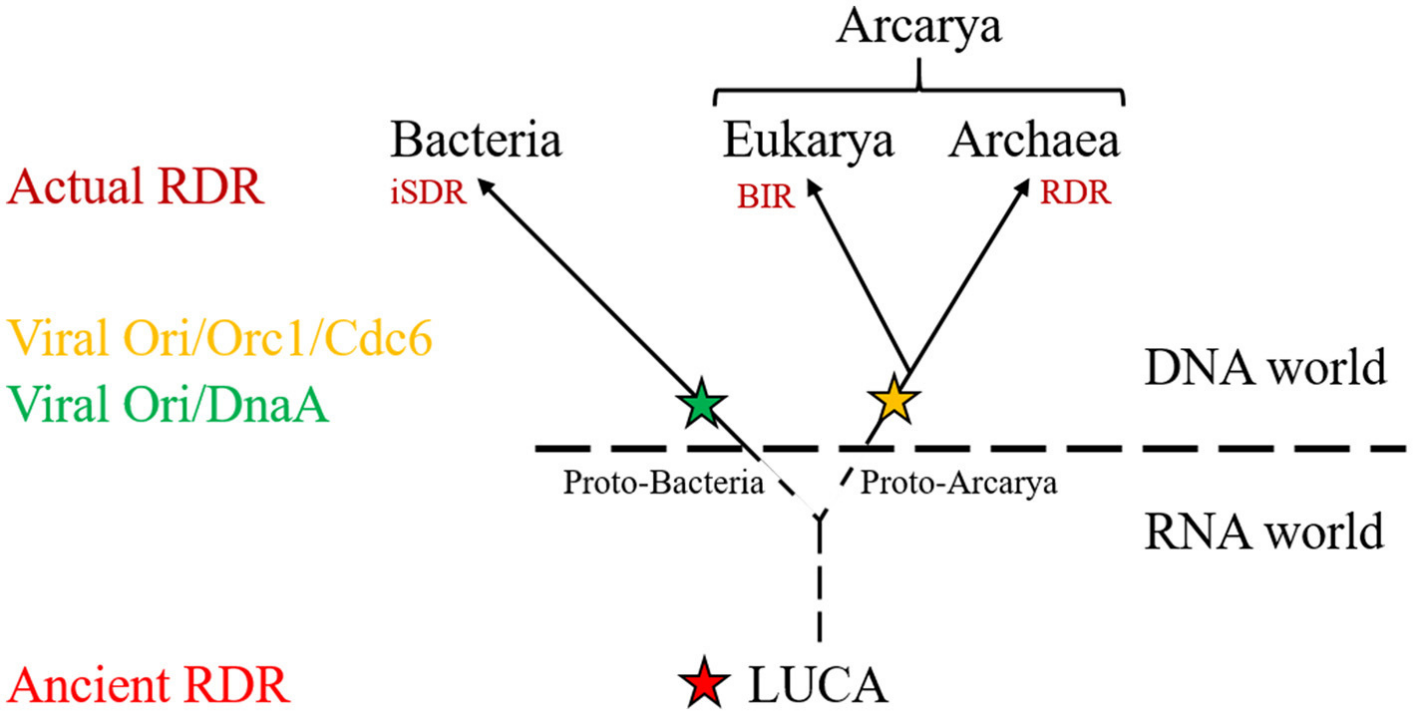
Schematic representation of the tree of life showing the distribution of RNA or DNA replication initiation pathway and their origins. Ancient RDR was transmit from LUCA to the three domain of life to generate actual RDR (BIR, RDR, iSDR). Viruses provided the origins of replication (Ori/DnaA; Ori/Orc1/Cdc6) in LUCA's descendance.

Whereas *ori* seems to be the main DNA replication system for *H. volcanii* (Hawkins et al., 2013), RDR appears to be the principal mechanism used to replicate DNA in *T. barophilus* and *T. kodakarensis* under laboratory conditions (Gehring et al., 2017; Mc Teer et al., 2024), as *ori* is mainly used during the stationary phase or not used at all except when RadA, the recombinase, is weakly expressed (Mc Teer et al., 2024; Liman et al., 2024). These findings underscore the versatility of archaeal replication, which appears far more complex and adaptable than the systems seen in bacteria. This adaptability, I believe, positions archaea as key models for understanding how early life forms evolved mechanisms to replicate their genomic DNA in extreme environments. Furthermore, these insights challenge the perception that prokaryotic replication is inherently simpler than eukaryotic processes. I propose that before the appearance of defined *ori*, early life forms might have relied on recombination-based processes, today mainly used for DNA repair, to replicate their genetic material (Figure 1). This reinforces the notion that evolutionary innovation often arises from the co-option of existing, often selfish, elements, as abundantly shown in scientific literature (Hurst and Werren, 2001; Hazen et al., 2010; Koonin, 2016; Haudiquet et al., 2022; Kumon and Lampson, 2022; Widen et al., 2023).

## 4 Conclusion

In conclusion, DNA replication in archaea represents a fascinating and complex area of study that bridges the gap between prokaryotes and eukaryotes. Recent studies, such as those by Hawkins et al. (2013), Gehring et al. (2017), Mc Teer et al. (2024), and Liman et al. (2024), highlight the unique and versatile nature of archaeal replication systems. These findings challenge long-standing assumptions and point to greater complexity in prokaryotic life than previously recognized. More effort should be directed toward studying other archaea to determine if only Euryarchaeota can use RDR. Badel and Bell (2024) mention that *Aeropyrum pernix*, like *T. barophilus*, uses *ori* more actively during the stationary phase, suggesting that even in some Crenarchaeota, RDR may be employed during the exponential phase. Additionally, analyzing the potential role of RecA in DNA replication in Cyanobacteria could provide further insights. Moreover, because Deinococcales are deeply branched in the tree of life and possess ESDSA (extended synthesis-dependent strand annealing; Zahradka et al., 2006), a specific Deinococcales double-strand break repair system, it would be interesting to test their capacity to live without *ori* or *dnaA*.

Archaea's ability to survive and replicate in extreme environments provides valuable models for understanding the limits of life on Earth and potentially other planets. By studying how archaea manage replication under high-stress conditions, we can better understand the evolutionary pressures that shaped early life forms.

Moving forward, interdisciplinary research integrating evolutionary biology, biochemistry, and biotechnology will be key to unlocking the full potential of these discoveries. Archaea are not only biological curiosities; they are central to our understanding of life's evolutionary history and hold promise for future biotechnological advancements.
